# Rapid Growth Reduces Cold Resistance: Evidence from Latitudinal Variation in Growth Rate, Cold Resistance and Stress Proteins

**DOI:** 10.1371/journal.pone.0016935

**Published:** 2011-02-24

**Authors:** Robby Stoks, Marjan De Block

**Affiliations:** Laboratory of Aquatic Ecology and Evolutionary Biology, University of Leuven, Leuven, Belgium; Northeastern University, United States of America

## Abstract

**Background:**

Physiological costs of rapid growth may contribute to the observation that organisms typically grow at submaximal rates. Although, it has been hypothesized that faster growing individuals would do worse in dealing with suboptimal temperatures, this type of cost has never been explored empirically. Furthermore, the mechanistic basis of the physiological costs of rapid growth is largely unexplored.

**Methodology/Principal Finding:**

Larvae of the damselfly *Ischnura elegans* from two univoltine northern and two multivoltine southern populations were reared at three temperatures and after emergence given a cold shock. Cold resistance, measured by chill coma recovery times in the adult stage, was lower in the southern populations. The faster larval growth rates in the southern populations contributed to this latitudinal pattern in cold resistance. In accordance with their assumed role in cold resistance, Hsp70 levels were lower in the southern populations, and faster growing larvae had lower Hsp70 levels. Yet, individual variation in Hsp70 levels did not explain variation in cold resistance.

**Conclusions/Significance:**

We provide evidence for a novel cost of rapid growth: reduced cold resistance. Our results indicate that the reduced cold resistance in southern populations of animals that change voltinism along the latitudinal gradient may not entirely be explained by thermal selection *per se* but also by the costs of time constraint-induced higher growth rates. This also illustrates that stressors imposed in the larval stage may carry over and shape fitness in the adult stage and highlights the importance of physiological costs in the evolution of life-histories at macro-scales.

## Introduction

Growth rate is a key life history trait that will determine age and size at maturity and therefore contributes in shaping adult fitness [1], [2]. Despite the obvious benefits of growing fast, i.e. reaching a large size in a short time, it is becoming widely accepted that animals typically are not growing at their maximum speed [3]–[5]. This has initiated a search for counterbalancing costs of growing fast with most focus on ecological costs like increased predation risk (e.g. ref [6]–[7]).

Physiological costs of rapid growth, i.e. those entailing a lower ability to endure adverse environmental conditions, are much less studied [8]. Most attention went to food shortage demonstrating that faster growing individuals did worse in coping with starvation (e.g. ref [9]–[11]). Although, it has been hypothesized that faster growing individuals would also do worse in dealing with suboptimal temperatures [8], this type of cost has never been explored empirically.

Costs as part of trade-offs can not only be studied at the individual but also at the population level because also interpopulation differences in growth rates exist. As the benefits and costs of rapid growth may vary among populations, optimal growth rates also show geographic variation [8]. In this context, time constraints associated with the length of the available growth period play an important role in shaping higher growth rates [12]. Changes in voltinism with more generations in low-latitude (-altitude) populations are widely documented along latitudinal (altitudinal) gradients [13]–[14], and have been identified as a key factor causing higher time constraints and therefore higher growth rates in these populations [15]–[16]. This generates the hypothesis that the reduced ability to deal with low temperatures in low-latitude (-altitude) populations (e.g. ref [16]–[17] may be partly explained as a physiological cost of the higher growth rates rather than by thermal selection *per se*.

The mechanistic basis of the physiological costs of rapid growth is largely unexplored. With regard to cold resistance, stress proteins like Hsp70 seem to play an important role in insects. Several studies showed an upregulation of Hsp70 under cold stress (e.g. ref [18]–[19]) which improves survival at low temperatures [20]–[21]. Also, chill coma recovery times, a key measure of cold resistance, have been linked to PGI genotypes [22], and PGI genotypes differ in Hsp70 expression [23]. Furthermore, higher Hsp70 levels are associated with reduced growth rates (overview in [24]). Altogether, this suggests that the proposed physiological cost of rapid growth in terms of reduced cold resistance may be mediated through reduced expression of Hsp70 in fast growers.

The overall aim of the present study is to evaluate physiological costs of rapid growth in terms of reduced cold resistance, as measured by chill coma recovery times, both at the individual and at the population level using the damselfly *Ischnura elegans* as a model system. At the population level we compared two northern univoltine with two southern multivoltine populations. Given the stronger time constraints associated with multivoltinism, we expected higher growth rates in the southern populations [12]. Based on previous empirical research (see above) we expected lower cold resistance in the southern populations, and rapid growth to be associated with reduced cold resistance potentially through a link with lower Hsp70 levels. Because patterns in growth rate and cold resistance among populations along latitudinal gradients may depend on rearing temperature [15], [17], [25], we reared larvae at three temperatures from the egg stage in a common-garden experiment. Clear latitudinal patterns in growth rate were observed. Higher growth rates were associated with reduced cold tolerance both at the population and at the individual level, and this relationship was consistent with differences in Hsp70 levels at the population level.

## Materials and Methods

The damselfly *Ischnura elegans* is a very abundant damselfly in Europe occurring from northern Spain to southern Sweden [26]. In northern Europe it has one generation a year (univoltine), while in southern Europe it has multiple generations a year (multivoltine) [13]. Two populations were studied from southern Sweden, Genarp (GE) and Vallby (VA), and two from southern France, St-Martin de Crau (SM) and Salin de Giraud (SG). To assess latitudinal differences in growth rate and cold resistance and their plastic responses to rearing temperature, we reared larvae of the four study populations from the egg stage until emergence at three temperatures (18°C, 21°C and 24°C) and measured chill coma recovery time one day after emergence in the adult stage. This temperature range has been shown to generate clear thermal reaction norms in related species [27] and spans the natural temperature regime of the populations of this species during the largest part of the growth season. Furthermore, survival is low when larvae are reared at lower and higher temperatures.

We collected 8–10 females for each of four study populations. Field-collected females were placed individually in small plastic containers and given wet filter paper as oviposition substrate and allowed to oviposit for three days in the laboratory. Afterwards they were released in the field. Rearing experiments were performed with the permission (09-06037) of the Flemish Agency of Nature and Forestry. Filter papers with eggs were transported to Belgium where they were kept at 21°C. At the day of hatching, larvae were randomly divided among six identical incubators set at 18°C, 21°C and 24°C (14∶10 L:D photoperiod). Each larva was placed individually in a circular plastic 180 ml cup filled to a height of 5 cm with aged dechlorinated tap water. Cups were rotated daily within the incubator and regularly between the incubators of the same temperature treatment. Larvae were daily fed *ad libitum* with brine shrimp nauplii. When larvae entered the final instar the daily food ration was doubled. We daily checked animals for adult emergence. Development time was calculated as the number of days between egg hatching and adult emergence. One day after emergence, each adult was weighed to the nearest 0.01 mg and randomly assigned to either a control or a cold shock treatment that induced a comatose condition. Larval growth rate was quantified as ln(mass at emergence)/development time (see e.g. ref [28]–[30]). This growth rate based on the entire larval period correlates strongly with growth rates based on mass increase during the final instar in this species (R. Stoks, unpublished data). For the life history variables, sample sizes per combination of latitude and temperature varied between 35 and 53 (total *n* = 261).

Cold resistance was measured as chill coma recovery time; an assay used successfully before to demonstrate intraspecific latitudinal and altitudinal patterns (e.g. ref [16]–[17], [31]–[32]). For this, we placed individual one-day old adults in a microcentrifuge tube in an incubator at 4°C at 11am. We chose this chilling temperature based on the cold challenges imposed on the natural adult populations (File S1). After 1.5 h each adult was gently placed on its back in a petri dish with roughened bottom at 21°C. We scored recovery times to the nearest second as the time taken for an animal to stand upright. Following recovery, animals were given another hour to allow for the possible upregulation of Hsp70 and were then frozen at −80°C (see ref [18]). No animals died during the cold shock. For recovery times, sample size per combination of latitude and temperature varied between 21 and 26 animals (total *n* = 140).

We quantified Hsp70 levels using an immunoblot assay closely following the protocol of Slos and Stoks [33]. Briefly, single larvae were homogenised in a proteinase inhibitor cocktail (Sigma®, St Louis, MO P2714) and a sample corresponding with 10 µg of protein was separated using SDS-polyacrylamide gel electrophoresis (PAGE). Therefore, any patterns in Hsp70 are independent of total protein content. Stress proteins were detected using monoclonal primary antibodies cross-reacting with the stress-induced Hsp70 and the constitutive Hsc70 (dilution 1∶1500, SPA 757, Stressgen®) and a AP-conjugated secondary antibody (dilution 1∶1000, D0486, DakoCytomation®, Glostrup, Denmark). The optical density (OD) of stress protein bands on the membrane was quantified on digitized images using the software package Image ProPlus. To correct for potential variation between blots, we ran a control sample of 1 µL HeLa Cell Lysate (Heat shocked; Stressgen®) on every blot. For Hsp70 the response curve for optical density against concentration is linear in damselflies [33]. For Hsp70, we analyzed per combination of latitude and temperature 11–13 control animals that received no cold shock and 22–26 animals that received a cold shock (total *n* = 212). Control animals for Hsp70 analysis were similarly treated as the ones given a cold shock but placed 1.5 h in an incubator at 21°C and not at 4°C. We assayed more larvae after the cold shock as our focus was on testing for covariation of Hsp70 levels and recovery times.

### Statistical analyses

We tested for effects of rearing temperature, latitude, and population nested in latitude on the dependent variables in separate general linear models. Population nested in latitude was included as a random factor; it was never significant (all *P*>0.23) indicating consistent results within a given latitude. In all analyses we also included sex and its interactions but these results are not related to out predictions and did not interfere with the observed patterns and therefore will not be reported. Models on life history (age and mass at emergence, growth rate) initially also included the cold shock treatment (present vs absent) to evaluate whether we successfully randomized larvae across the two adult cold shock treatments. Yet, it was never significant (all *P*>0.13) and not retained in the final models. The cold shock treatment was also included as a factor when analyzing Hsp70 levels but not when analyzing recovery times because for the latter variable all animals had been given a cold shock. For the analyses of recovery times (log-transformed) and Hsp70 levels we included mass as a covariate, and for the latter also the optical density of the Hela control. Correct degrees of freedom were estimated using the Satterthwaite option. Because the results on larval development time ( =  age) and mass at emergence are not the focus of this paper they are presented in File S2 and Figure S1.

## Results

**Figure 1:**
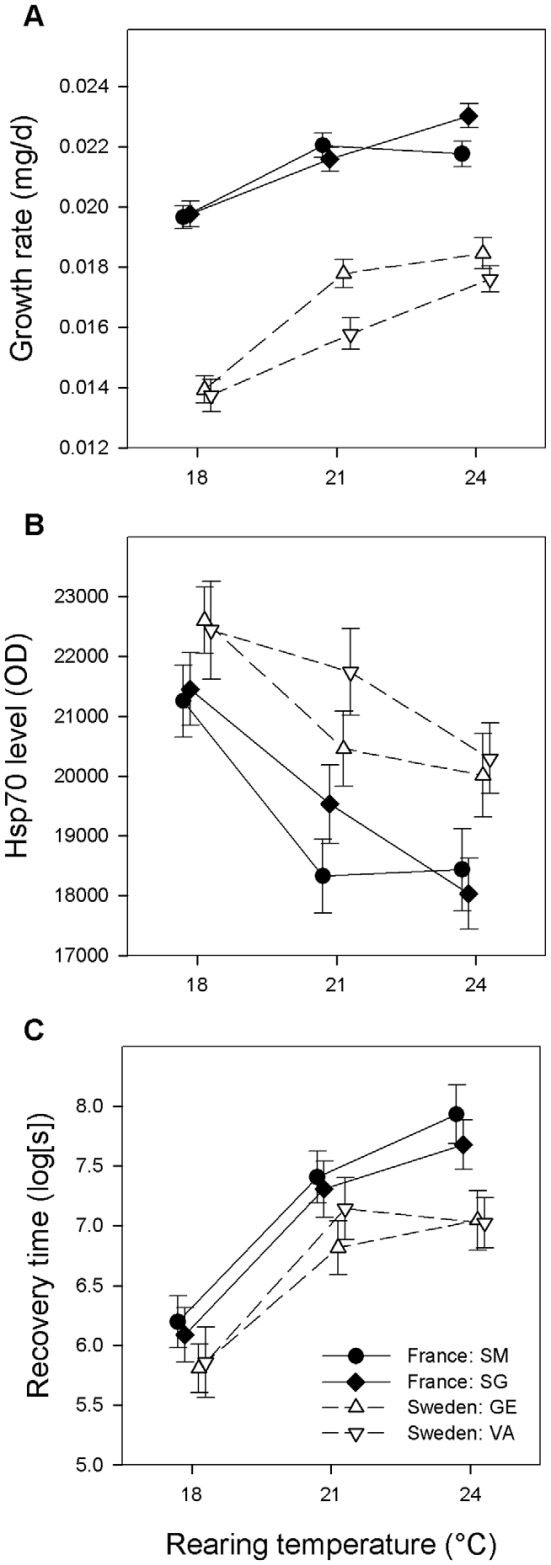
Differences in growth rate, Hsp70 level and chill coma recovery time between latitudes across temperatures. Mean (±1 SE) larval growth rate (A), and Hsp70 level (B) and chill coma recovery time (C) in the adult stage of *Ischnura elegans* from two northern and two southern populations at three rearing temperatures. Means are slightly offset to aid visualization. Hsp70 levels and chill coma recovery times are quantified after a cold shock treatment (1.5 h exposure to 4°C) given to the freshly emerged adults.

Southern larvae had much higher growth rates than northern larvae (*F*_1,1.81_ = 123.13, *P* = 0.011; Fig. 1A). With increasing temperature growth rate increased (*F*_2,248_ = 54.67, *P*<0.0001). This temperature-induced plasticity in growth rate was less pronounced in southern larvae (Latitude × Temperature, *F*_2,248_ = 5.66, *P* = 0.0039).

**Figure 2:**
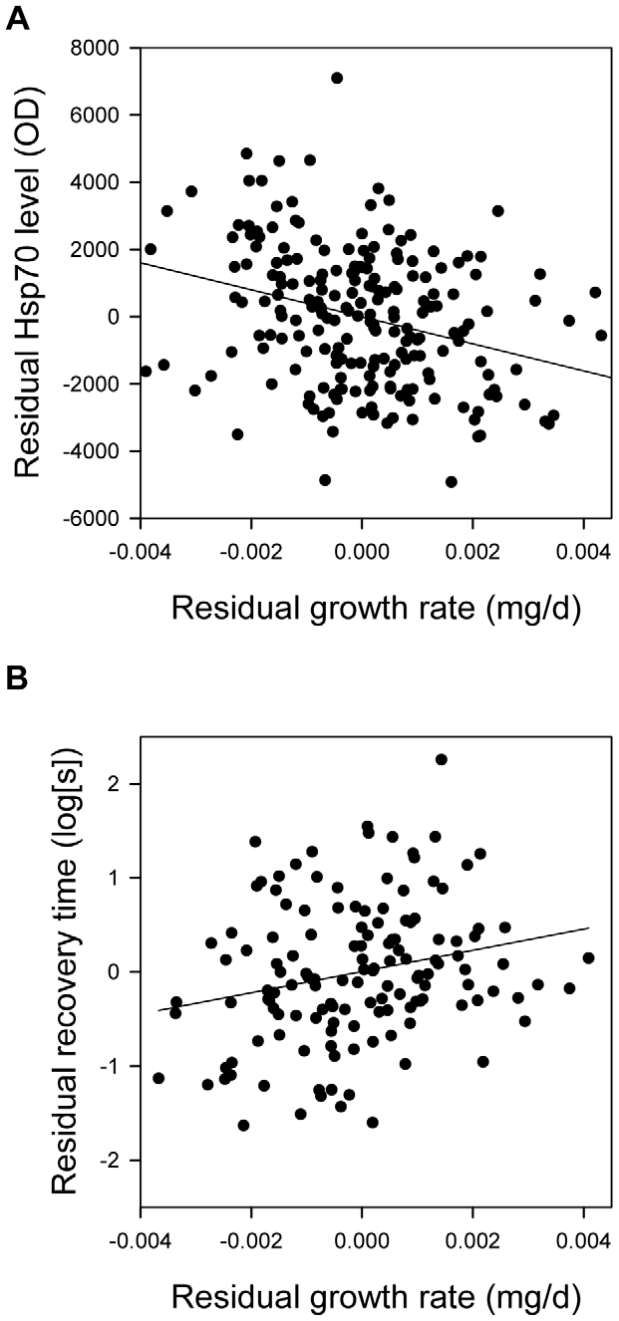
Relationships between growth rate, Hsp70 level and chill coma recovery time. Relationships between larval growth rate and residual model values of (A) Hsp70 level and (B) chill coma recovery time in the adult stage of *Ischnura elegans*. Residuals were derived from the general models described in the methods and therefore independent of temperature and latitude.

After a cold shock, Hsp70 levels were slightly lower (*F*_1,186_ = 5.59, *P* = 0.019; mean OD ±1SE, without cold shock: 20,800±320, with cold shock: 20,100±210). Hsp70 levels were lower in southern animals (*F*_1,186_ = 4.28, *P* = 0.040) and especially the rearing temperature effect was strong with considerably lower Hsp70 levels at the higher temperature (*F*_2,186_ = 25.80, *P*<0.0001) (Fig. 1B). When adding growth rate to the model the latitude (*F*_1,20.9_ = 7.84, *P* = 0.011) and temperature (*F*_2,180_ = 5.80, *P* = 0.0036) effects remained; larvae with a higher growth rate had lower Hsp70 levels (*F*_1,178_ = 23.90, *P* = 0.040, *R^2^* = 0.09; Fig. 2A).

Recovery times were longer in southern animals (*F*_1,127_ = 8.27, *P* = 0.0047) and in animals reared at higher temperatures (*F*_2,127_ = 38.64, *P*<0.0001) (Fig. 1C). When also including larval growth rate and Hsp70 levels in the model, the latitudinal effect was no longer significant (*F*_1,10.6_ = 1.03, *P* = 0.33) while the temperature effect remained significant (*F*_2,124_ = 10.97, *P*<0.0001). Larvae with a higher growth rate had longer recovery times (*F*_1,123_ = 9.71, *P* = 0.0023, *R^2^* = 0.06; Fig. 2B). Hsp70 levels did not affect recovery times (*F*_1,124_ = 0.63, *P* = 0.43).

## Discussion

In line with the higher perceived time constraints [6]–[7], [11]–[12], [34], growth rates were higher in the southern multivoltine than in the northern univoltine populations. Similar latitudinal growth rate patterns associated with changes in voltinism have been documented in butterflies [35], mosquitoes [15], and in a previous study on *I. elegans* (Shama et al., unpublished data). We also found the typical increase in growth rates at higher temperatures [36]. This increase was less pronounced in the southern populations where growth rates were already high at the low temperature, suggesting growth rates were near their physiological maximum [11].

We provide evidence of a novel cost of rapid growth: rapid growth was associated with a reduced cold resistance both at the latitudinal level and at the individual level. Importantly, these patterns cannot simply result from differences in mass, as all analyses were mass-corrected.

More generally, our results support the untested hypothesis by Gotthard [8] that faster growing individuals would be worse in dealing with suboptimal temperatures. In ectotherms like damselflies that forage and mate in flight, there are obvious fitness implications of a higher cold resistance in the adult stage. Adults with a better cold resistance would better endure cold nights and likely be active earlier in the day, hence can spend more time foraging and engaging in reproductive activities [37]. This cost may be general in animals and plants, yet not directly considered in previous studies. As in our study, southern populations of the pitcher plant mosquito have higher growth rates [15] and a lower cold resistance than northern populations [17]. Further, lowland populations of the copper butterfly have higher growth rates and reduced cold resistance [16]. Finally, faster growing plant species have higher frost damage [38].

Cold resistance, as measured by shorter chill coma recovery times, was higher in the northern populations and higher at the lowest rearing temperature. This is in line with studies along latitudinal gradients in other insects (e.g. ref [17], [31]–[32]), which considered this latitudinal pattern as a direct result of geographic differences in thermal selection. Also in our study system thermal selection for increased cold resistance is likely higher in the northern populations (File S1). Yet, the observation that latitudinal differences in cold resistance were not significant anymore when growth rate was added to the model indicates growth rate differences are contributing to the latitudinal differences in cold resistance. We therefore hypothesize that the latitudinal pattern in cold resistance in our study system may not be entirely explained by thermal selection *per se* but also by the higher growth rates at lower latitudes.

While there was some indication that the physiological cost of rapid growth in terms of reduced cold resistance was mediated through reduced Hsp70 levels there was no support for this at the individual level. Treatment groups with higher growth rates and longer recovery times, i.e. southern populations and animals reared at higher temperatures, indeed had lower Hsp70 levels, and at the individual level faster growing animals had lower Hsp70 levels. Yet, individual variation in Hsp70 levels did not explain variation in recovery times when taking into account the treatment effects. A reason for this may be that we could not detect an upregulation of Hsp70 under cold stress and that we therefore obtained baseline Hsp70 levels (see below). More general, other (stress) proteins may also play a role in shaping the still poorly understood resistance to nonfreezing low temperatures [39]–[40], potentially obscuring any effect, if present, from Hsp70 alone.

Instead of an upregulation of Hsp70 after the cold shock, we found a slight downregulation. In the copper butterfly Karl et al. [18] did report an upregulation of Hsp70 after a 1 h cold stress followed by a 1 h recovery period. Yet, in line with our results, other studies found that (mRNA) levels of Hsp70, if anything, decreased during a cold period and only started increasing several hours after the cold shock ended [19], [21], [41]. Activation of the heat shock factors is probably incomplete under a short period of cold stress, and longer recovery times would have shown an upregulation [19], [21]. Whatever the reason, this lack of upregulation during the experiment indicates that baseline Hsp levels (i.e. those present before the cold shock) may have contributed in shaping the latitudinal differences in recovery times in current experiment. Upregulation of Hsp70 after cold stress may be more important in reducing damage long after the chill coma has ended as shown in another insect [21] and may not be that important in keeping chill coma recovery times short. Similarly, Koštál and Tollarová-Borovanská [21] suggested that high Hsp levels during dormancy may represent an anticipatory protection against a variety of environmental insults.

Hsp70 levels were lower in the two treatment groups with the highest growth rates, southern larvae and larvae reared at the high temperature, consistent with an energetic cost of rapid growth [11]. Importantly, also at the individual level faster growing larvae had lower Hsp70 levels. Note that, development time (nor body mass) did not covary with Hsp70 levels when also growth rate was included in the model, so it is not a faster life history (or associated patterns in body mass) *per se* that seems to shape Hsp70 patterns. This trade-off pattern between growth rate and Hsp70 levels adds to the few other studies showing that higher Hsp levels were associated with lower growth rates (overview in [24]). For example, *Drosophila* larvae with extra copies of the Hsp70 gene have decreased growth rates compared to control larvae [42]. This trade-off is thought to be energy-mediated as the synthesis, functioning and maintenance of Hsp proteins is energetically costly [24].

To conclude, we here presented evidence for a, likely widespread, novel cost of rapid growth in terms of reduced cold resistance. Our study thereby is complementary to the few other studies demonstrating physiological costs of rapid growth in terms of reduced resistance against food stress (see introduction), oxidative stress [43]–[44] and reduced immune function [45]–[46] and offers a new explanation why organisms typically not grow at their maximal rates [3]–[5]. Noteworthy, this type of cost would never have been detected when only focusing on the adult stage, stressing the importance to consider both life stages when trying to understand life history variation in animals with a complex life cycle [47]. This adds to the insight that stressors imposed in the larval stage may carry over and shape fitness in the adult stage [48]–[49]. Furthermore, this type of cost may also contribute to the here documented latitudinal patterns in cold resistance. Changes in voltinism with more generations in low-latitude (-altitude) populations are widely documented [13]–[14], and have been identified as a key factor generating higher time constraints and therefore higher growth rates in these populations [15]–[16]. Together with current findings this may indicate that the reduced ability to deal with low temperatures in low-latitude (-altitude) populations of animals that change voltinism along these gradients (e.g. ref [16]–[17]) may not be entirely explained by thermal selection *per se* but also by the costs of the time constraint-induced higher growth rates. Our results thereby highlight the importance of physiological costs in the evolution of life-histories at macro-scales. Given that cold resistance is a key factor shaping range limits, similar studies at the interplay between physiological ecology and macro-ecology may prove rewarding in understanding range boundaries and their shifts under global warming [50]–[51].

## Supporting Information

File S1 **Motivation chill coma temperature.**
(DOC)

File S2 **Effects of latitude and temperature on age and mass at emergence.**
(DOC)

Figure S1 **Differences in development time and mass at emergence between latitudes across temperatures.** Mean (±1 SE) larval development time (A), and mass at emergence (B) of Ischnura elegans from the two northern and two southern populations at the three rearing temperatures. Means are slightly offset to aid visualization.
(TIF)
