# Supplementary material for: Rapid Growth Reduces Cold Resistance: Evidence from Latitudinal Variation in Growth Rate, Cold Resistance and Stress Proteins
Source: PLoS One. 2011 Feb 24;6(2):e16935. doi: 10.1371/journal.pone.0016935 (PMC3044720; doi:10.1371/journal.pone.0016935)
Supplement: File S1 — Motivation chill coma temperature. (DOC) [file pone.0016935.s001.doc]

**Supporting information file 1.**

**Motivation chill coma temperature.**

Daily minimum air temperature data were compared between latitudes for the period 1990-2008. Data from southern Sweden were collected at Malmö by the Swedish Meteorological Institute (SMHI). Data from southern France were collected at the research institute Tour-du-Valat (Le Sambuc) by Philippe Chauvelon. Both weather stations are within 30 km from the study populations.

During the 19 yr recording period, air temperatures throughout the respective (terrestrial) adult flight periods never went down to 4°C in southern France yet reached 4°C for in total 70 days in southern Sweden. During winter temperatures experienced in the aquatic larval stage typically not go below 4°C under the ice [1]. Across the entire year, so including the larval growth period, temperatures reached 0°C (hence, probably 4°C under the ice), about 10 weeks per year in southern Sweden and about three weeks per year in southern France. Therefore, to link our measure of cold resistance to realistic levels experienced in the field [2], we decided to give a cold shock at 4°C. Pilot trials showed that adults readily entered chill coma at this temperature and that recovery time patterns in function of latitude and rearing temperature were shorter, yet similar to those obtained after a cold shock at 0°C. Furthermore, Hsp70 has been shown to be upregulated at 4°C in other insects (e.g. ref [3]).

**References**

1. De Block M., McPeek MA, Stoks R (2007) Winter compensatory growth under field conditions partly offsets low energy reserves before winter in a damselfly. Oikos 116:1975-1982.
2. Hoffmann AA, Sørensen JG, Loeschcke V (2003) Adaptation of *Drosophila* to temperature extremes: bringing together quantitative and molecular approaches. J Therm Biol 28:175-216.
3. Zhang QR, Denlinger DL (2010) Molecular characterization of heat shock protein 90, 70 and 70 cognate cDNAs and their expression patterns during thermal stress and pupal diapause in the corn earworm. J Ins Physiol 56:138-150.
