# Supplementary material for: Rapid Growth Reduces Cold Resistance: Evidence from Latitudinal Variation in Growth Rate, Cold Resistance and Stress Proteins
Source: PLoS One. 2011 Feb 24;6(2):e16935. doi: 10.1371/journal.pone.0016935 (PMC3044720; doi:10.1371/journal.pone.0016935)
Supplement: File S2 — Effects of latitude and temperature on age and mass at emergence. (DOC) [file pone.0016935.s002.doc]

**Supporting information file 2.**

**Effects of latitude and temperature on age and mass at emergence.**

Southern larvae had much shorter development times than northern larvae (*F*_1,1.87_ = 112.30, *P* = 0.011; Fig. S1A). With increasing temperature development times decreased (*F*_2,248_ = 106.92, *P* < 0.0001). This temperature-induced plasticity in development was less pronounced in southern larvae (Latitude × Temperature, *F*_2,248_ = 23.22, *P* < 0.0001).

Southern larvae were somewhat smaller than northern larvae, yet this was not significant (*F*_1,2.41_ = 7.06, *P* = 0.09; Fig. S1B). With increasing temperature mass at emergence decreased (*F*_2,248_ = 11.42, *P* < 0.0001). This temperature-induced plasticity in mass did not differ between latitudes (Latitude × Temperature, *F*_2,248_ = 1.48, *P* = 0.23).
