# Supplementary figures and images for: Rapid Growth Reduces Cold Resistance: Evidence from Latitudinal Variation in Growth Rate, Cold Resistance and Stress Proteins
Source: PLoS One. 2011 Feb 24;6(2):e16935. doi: 10.1371/journal.pone.0016935 (PMC3044720; doi:10.1371/journal.pone.0016935)

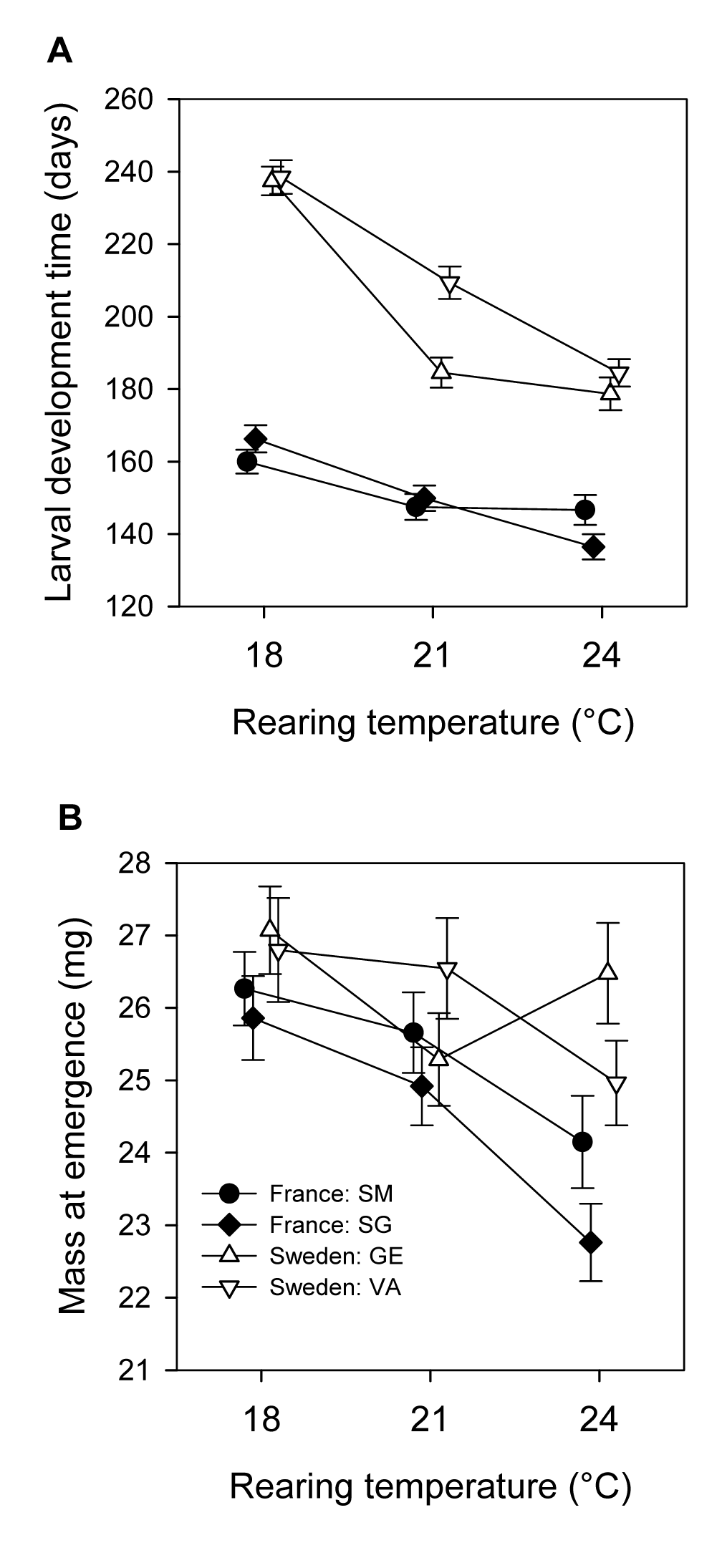

Supplement: Figure S1 — Differences in development time and mass at emergence between latitudes across temperatures. Mean (±1 SE) larval development time (A), and mass at emergence (B) of Ischnura elegans from the two northern and two southern populations at the three rearing temperatures. Means are slightly offset to aid visualization. (TIF) [file pone.0016935.s003.tif]
